# Encapsulation of Thermo-Sensitive Lauric Acid in Silica Shell: A Green Derivate for Chemo-Thermal Therapy in Breast Cancer Cell

**DOI:** 10.3390/molecules24112034

**Published:** 2019-05-28

**Authors:** Valeria De Matteis, Mariafrancesca Cascione, Maria Luisa De Giorgi, Stefano Leporatti, Rosaria Rinaldi

**Affiliations:** 1 Dipartimento di Matematica e Fisica “E. De Giorgi”, Università del Salento, Via per Arnesano, 73100 Lecce, Italy; mariafrancesca.cascione@unisalento.it (M.C.); marialuisa.degiorgi@le.infn.it (M.L.D.G.); 2 CNR Nanotec-Istituto di Nanotecnologia, via Monteroni, c/o Campus Ecotekne, 73100 Lecce, Italy; stefano.leporatti@nanotec.cnr.it

**Keywords:** nanoencapsulation, hyperthermal therapy, lauric acid, breast cancer

## Abstract

Lauric acid is a green derivate that is abundant in some seeds such as coconut oil where it represents the most relevant fatty acid. Some studies have emphasized its anticancer effect due to apoptosis induction. In addition, the lauric acid is a Phase Change Material having a melting temperature of about 43.2 °C: this property makes it a powerful tool in cancer treatment by hyperthermal stress, generally induced at 43 °C. However, the direct use of lauric acid can have some controversial effects, and it can undergo degradation phenomena in the extracellular environment. For this reason, we have encapsulated lauric acid in a silica shell with a one-step and reproducible synthetic route in order to obtain a monodispersed SiO_2_@LA NPs with a good encapsulation efficiency. We have used these NPs to expose breast cancer cell lines (MCF-7) at different concentrations in combination with hyperthermal treatment. Uptake, viability, oxidative stress induction, caspases levels, and morphometric parameters were analyzed. These nanovectors showed double action in anticancer treatments thanks to the synergic effect of temperature and lauric acid activity.

## 1. Introduction

Fatty acids are the most important elements constituting phospholipids, triglycerides and cholesterol esters, representing an important dietary intake of energy [1]. Many plants that produce oils such as palm, coconut and olive are rich in fatty acids [2]. Among different fats, that are classified based on carbon atom numbers, lauric acid (LA) is saturated Medium Chain Fatty Acids (MCFA) having 12 carbon atoms. It is abundant in some fruits, breast milk, and in coconut oil [3]. Coconut oil is the major reservoir of LA constituting about the 50% of total fatty acids; it exhibits antibacterial activity [4,5], anti-inflammatory [6,7], anti-diabetes properties [8,9], and it is also used as topic agent in skin diseases [10,11]. Indeed, some studies highlighted the antitumor effect in cancer cells: Salerno et al. [12] finded the dramatic growth inhibition of HT-29 malignant human colon cell line induced by some oils including coconut. Lappano et al. [13] demonstrated the anti-proliferative and pro-apoptotic action of LA on breast and endometrial cancer cells in different ways. In particular, LA induced the ROS production together with up-regulation of the cyclin-dependent kinase inhibitor p21Cip1/WAF1. Similar effects were found in Colon Cancer Cells (CRC). Fauser et al. [14] compared butyrate and LA following their anticancer properties. They demonstrated that LA elicited S and G2/M phases arrest in Caco-2 and IEC-6 cell lines in a strong manner compared with butyrate. The same trend was observed after the Reactive Oxygen Species (ROS) and Glutathione (GSH) measurements: LA promoted high levels of ROS production compared to butyrate that protected IEC-6 cells from oxidative stress injury. For these reasons, LA can be considered an anticancer factor stimulating adverse effects in cancer cells. In addition, LA is classified as organic Phase Change Material (PCM) [15]. PCMs are substances that absorb and release thermal energy during the process of melting and freezing. In fact, they undergo a phase transition when the temperature reaching specific melting point: this is a physical property of each PCM material [16]. In particular, LA with a melting temperature of ~43 °C and a phase transition enthalpy (Δh) of ~184 kJ/kg [17] make it a powerful chemotherapeutic agent in combination with thermotherapy. The latter is a technique that permits to induce a hyperthermal stress (working temperature is in the range of 41–45 °C) in tumor site and, today, is the most widespread method to treat tumors (in particular breast cancer) without using surgery [18]. Contrary to chemotherapeutic agents, thermotherapy is low toxic and it is tolerable for most patients. In fact, it is simultaneously used with conventional chemical drugs responsible to eliminate the eventual residual tumor cells survived to high temperature [19]. In this study, novel nanocapsules consisting of a LA core encapsulated in silica shell (SiO_2_@LA NPs) were synthetized with a simple and reproducible chemical route. Among different materials, amorphous silica were chosen due to its high thermal conductivity, heat storage capacity and non-toxic behavior for living organisms [20]. Therefore, the encapsulation protected LA from degradation phenomena that could occur in living organisms. These nanomaterials were particularly suitable to trigger breast cancer cells lines (MCF-7) disruption by thermal induction at 43 °C (melting temperature of LA). In this way, the MCF-7 cell lines were doubly perturbed both for the high temperature and by the action of LA that operated as a chemotherapy agent inducing oxidative stress, apoptosis, and morphometric alterations.

## 2. Results and Discussion

**Figure 1:**
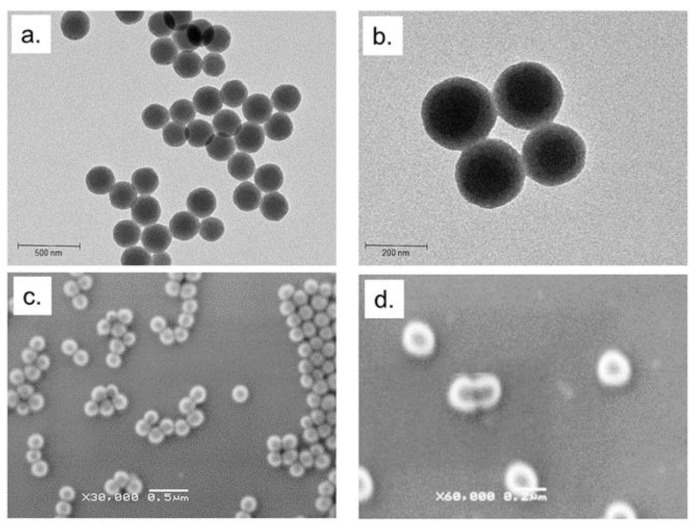
(**a**,**b**) Representative TEM images (acquired in bright field) and (**c**,**d**) SEM images of SiO_2_@LA NPs (acquired by secondary electrons).

Breast cancer is one of the most common types of cancer for women and, despite important progresses being made in therapy strategies, it remains one of the most fatal diseases [21,22]. The current golden standard treatment combines the use of chemotherapeutic drugs with mastectomy or radiotherapy [23]. Nevertheless, the drugs are often toxics and a lot of collateral effects are observed due to their a-specific action [24]. Thermotherapy is a useful physical method in the tumor treatment due to the use of high temperature (about 43 °C) to destroy malignant cells [25]. This strategy has different advantages such as low toxicity, absence of collateral effects and high tolerability of patients [26,27]. In general, this method is used in combination with conventional treatment such as drugs. In recent years, the study of green derivates has shown their potential anticancer activity [28,29]. Previous studies have focalized the role of fatty acids in several transduction pathways and in fundamental biological events such as apoptosis, inflammation, and induction of bioactive lipid mediators production leading to conceive different patho-physiological responses [30,31,32,33]. Among these, LA, a saturated MCFA, induce several collateral effects in cancer cells [34]. In addition, its thermal properties make it a strong agent in the combined treatment with thermal therapy [35]. We explored the double action of LA encapsulating it in a silica shell by means of easy and reproducible one-step synthetic route. SiO_2_@LA NPs were firstly characterized by using TEM and SEM. In TEM acquisitions, NPs appeared spherical and monodispersed with a size of (220 ± 2) nm. (Figure 1a,b).

**Figure 2:**
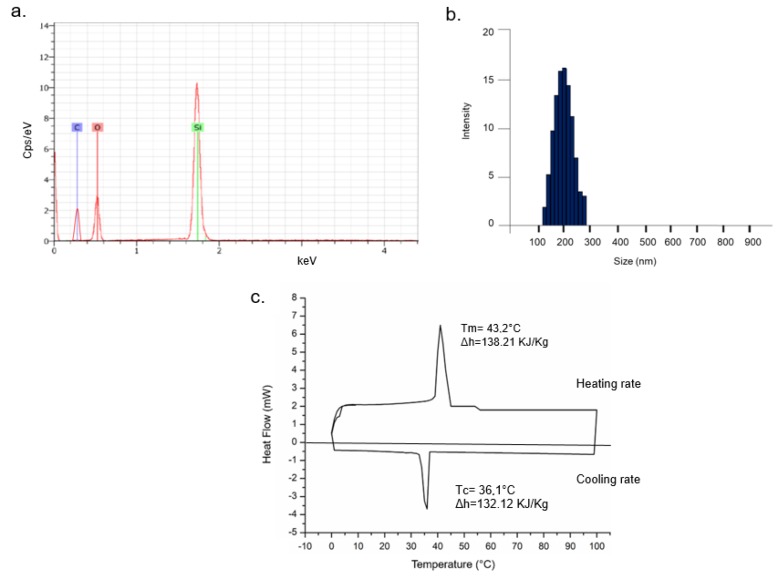
Energy-dispersive X-ray spectroscopy (EDS) analysis (**a**), dynamic light scattering (DLS) measurement (**b**) and DSC curve (**c**) of SiO_2_@LA NPs.

SEM images (Figure 1c,d) confirmed the morphology and surface of NPs whereas EDS analysis showed the presence of confined LA in the SiO_2_ core due to the presence of the silicon, oxygen and carbon element peaks in the graph (silicon and oxygen for silica and carbon and oxygen for LA) (Figure 2a). Monodispersion and size were also confirmed in solution by DLS, showing a diameter of 230 ± 3 nm (Figure 2b). In DMEM, the size increased due the formation of protein corona (250 ± 5) nm (data not shown) [36]. Thermal stability of SiO_2_@LA NPs was studied by mean DSC that showed the heating rate (between −10 °C and 100 °C at 1 °C/min) and the cooling rate (from 100 °C to −10 °C at 1 °C/ min) (Figure 2c).

Solid–liquid melting peak (that referred to the positive region of graph) and liquid–solid solidification peak (that referred to the negative region of graph) highlighted the absorbing heat process and releasing heat process, respectively. Then, the melting (Tm) and cooling (Tc) temperatures were measured to 43.2 °C and 36.1 °C. The melting enthalpy (∆hm) of encapsulated PCMs measured by DSC software (just integrate the area under the endothermic curve) reaches a maximum of 138.21 KJ/Kg whereas the ∆hm of LA in pure state is 184.29 KJ/Kg [17]. The cooling enthalpy (∆hc) was 132.12 KJ/Kg.

The encapsulation efficiency (R) is the percentage of drug (in this case LA) that was successfully entrapped into SiO_2_NPs. This value was calculated using the results obtained by DSC analysis (as the material was sensitive to heat) showing a Tm of ~ 43 °C. R was defined as follows:

(1) $$R=\frac{\Delta hm}{\Delta hmpcm}*100$$

where ∆hm is the specific melting enthalpy of NPs and ∆hmpcm was the specific melting enthalpy of the PCM in pure state: then, the resulting R was 75%.

The loading content of LA in the SiO_2_ core was detected by HPLC after the esterification of the fatty acid. The SiO_2_@LA NPs concentration was obtained weighing them after freeze drying process. In Table 1, the LA loading content was reported. Values were obtained by the equation described in the materials section.

**Figure 3:**
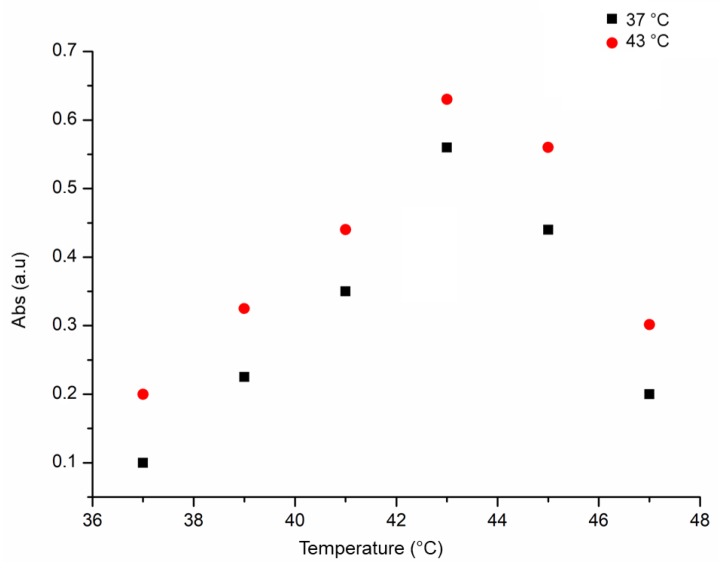
Thermo sensibility of SiO_2_@LA NPs (10 and 40 µg/mL) measured at different temperatures (37 °C, 39 °C, 41 °C, 43 °C, 45 °C, 47 °C).

The thermo sensibility of SiO_2_@LA NPs was detected monitoring the LA release in vitro at different temperature points with UV-vis analysis at 250 nm. As showed in Figure 3, the maximum absorbance of SiO_2_@LA NPs was recorded principally at 43 °C: this result confirmed their suitable application for thermotherapy.

**Figure 4:**
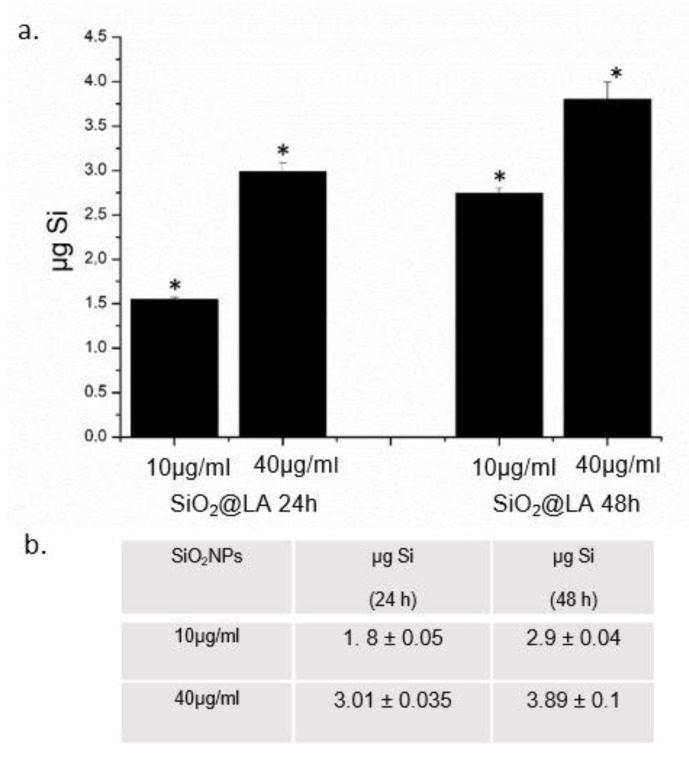
**a**. SiO_2_@LA NPs accumulation in MCF-7 cell lines exposed to 10 µg/mL and 40 µg/mL of NPs for 24 h and 48 h. Cells were then harvested, live cells were counted and Si content was measured in 360,000 cells (μg Si). **b**. blank SiO_2_NPs (10 µg/mL and 40 µg/mL) uptake in MCF-7 expressed as µgSi after 24 h and 48 h Data reported as mean ± SD from three independent experiments; statistical significance of exposed cells vs. control cells for *p* value ˂ 0.05 (<0.05 *).

The encapsulation route prevented the degradation and dispersion of this fatty acid: in this way the combination of hyperthermal stress and LA fusion/ release in the tumor cells has been demonstrated in MCF-7 cells. As a preliminary study, the quantification of the uptake levels of SiO_2_@LA NPs was analyzed. We carried out ICP-AES elemental analysis over cell lysate, after incubating MCF-7 cells with 10 µg/mL and 40 µg/mL of NPs for 24 and 48 h showing a time and concentration dependent uptake internalization (Figure 4a). In particular, after 48 h ca. 2.8 µg of Si were measured after a cells exposure of 10 µg/mL, whereas at the high concentration the levels of Si reached 3.7 µg. These values indicated the remarkable internalization of nanomaterials inside cells. Figure 4b reported the cells uptake values of blank SiO_2_NPs: significant differences in cell internalization did not recorded because the LA was encapsulated and the only interaction with plasma membrane occur with SiO_2_ shell.

**Figure 5:**
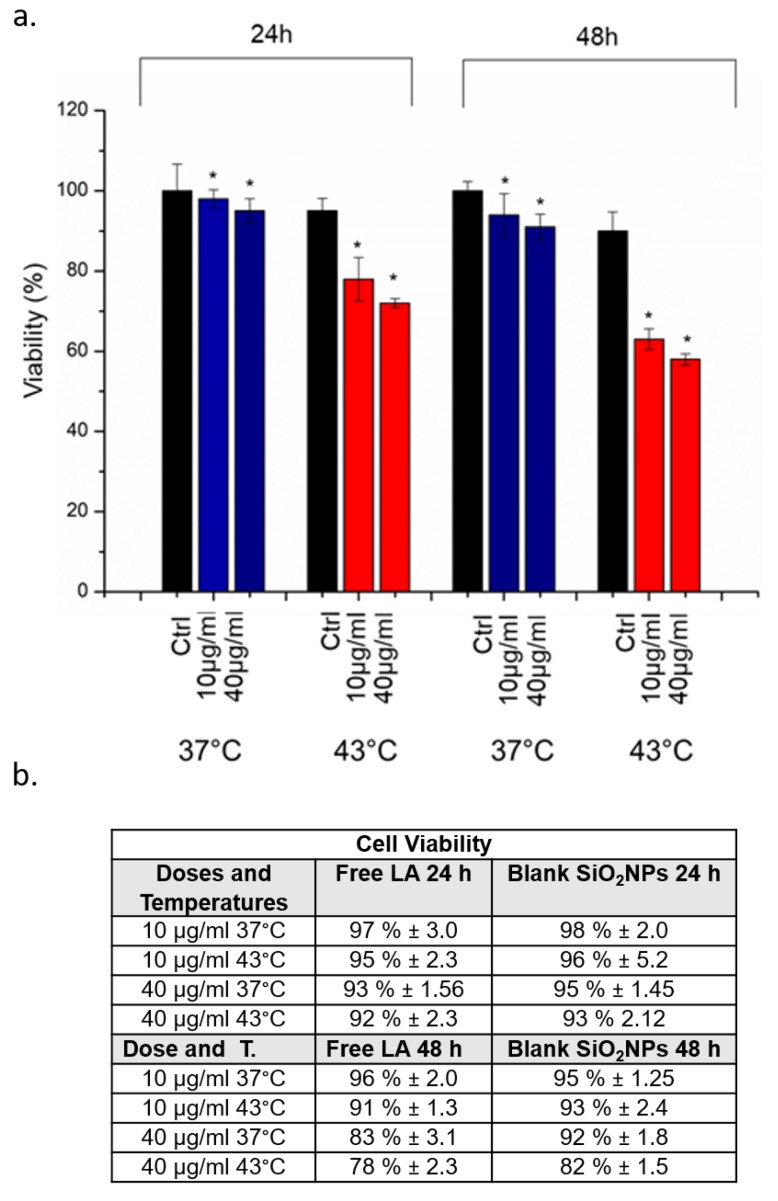
(**a**)Viability assay (WST-8) of MCF-7 cells after 24 h and 48 h exposure to 10 µg/mL and 40 µg/mL of SiO_2_@LA NPs at 37 °C and 43 °C. Percent viability of NP-treated cells was expressed relative to non-treated control cells (without SiO_2_@LA NPs exposure). As positive control (P), cells were incubated with 5% DMSO showing a ~ 60% viability decrease (data not shown). Data reported as mean ± SD from three independent experiments are considered statistically significant compared with control (*n* = 8) for *p* value ˂ 0.05 (<0.05 *). (**b**) Viability values expressed in percentages obtained on MCF-7 cells after 24 h and 48 h exposure to 10 µg/mL and 40 µg/mL of blank SiO_2_NPs and free LA at 37 °C and 43 °C.

After characterization and the uptake study, the effect of SiO_2_@LA NPs at 37 °C and 43 °C by hyperthermal stress induction in terms of viability, ROS production and caspase-3/caspase-9 induction were investigated. In general, the local hyperthermia treatment is conducted for 20–60 min in a temperature range of 40–45 °C [37,38]: in our work, we incubated cells for 45 min at 43 °C without and with SiO_2_@LA NPs at two concentrations (10 µg/mL and 40 µg/mL) and two time points (24 h and 48 h) in order to test the synergic activity of temperature and LA. The controls samples were represented by cells without SiO_2_@LA NPs exposure, but only exposed to 37 °C (physiological temperature) and 43 °C (thermal treatment temperature) at 24 h and 48 h: a good tolerance of cells to 43 °C at the two time points was observed. These evidences demonstrated that that the only temperature treatment did not affect the reduction of viability in strong manner (Figure 5a). In order to underline how the enhancement of biological effects was due to the LA nanoencapsulation; we performed the viability test on MCF-7 with the same procedure described in the section materials using free LA and blank SiO_2_NPs (Figure 5b) as further controls. The obtained values demonstrated a reduction of viability similar to the controls showed in Figure 5a (represented only by cells exposed to different temperatures). This effect can explained with the expression of Heat Shock Proteins (HSP) that are designate to repair the denatured proteins; they are also overexpressed in cancer cells due to their critical role in the proliferation process [39,40]. The same effects was observed when the cells were incubated with two concentrations of SiO_2_@LA NPs: at 37 °C, the NPs did not induced a significant decrease in terms of viability because the amorphous silica shell is low toxics for cells [41]. Therefore, the LA confined in the core was solid at 37 °C and, as a consequence, no release was shown. On the contrary, a higher reduction of viability was showed when high temperature and exposure to SiO_2_@LA NPs were combined: the double action induced a reduction of cellular viability respect to control in a dose dependent manner suggesting the role of LA as chemotherapeutic agent. In particular, the incubation with 40 µg/mL of NPs at 43 °C, only the 65% and 58% of cells were vital after 24 h and 48 h, respectively.

**Figure 6:**
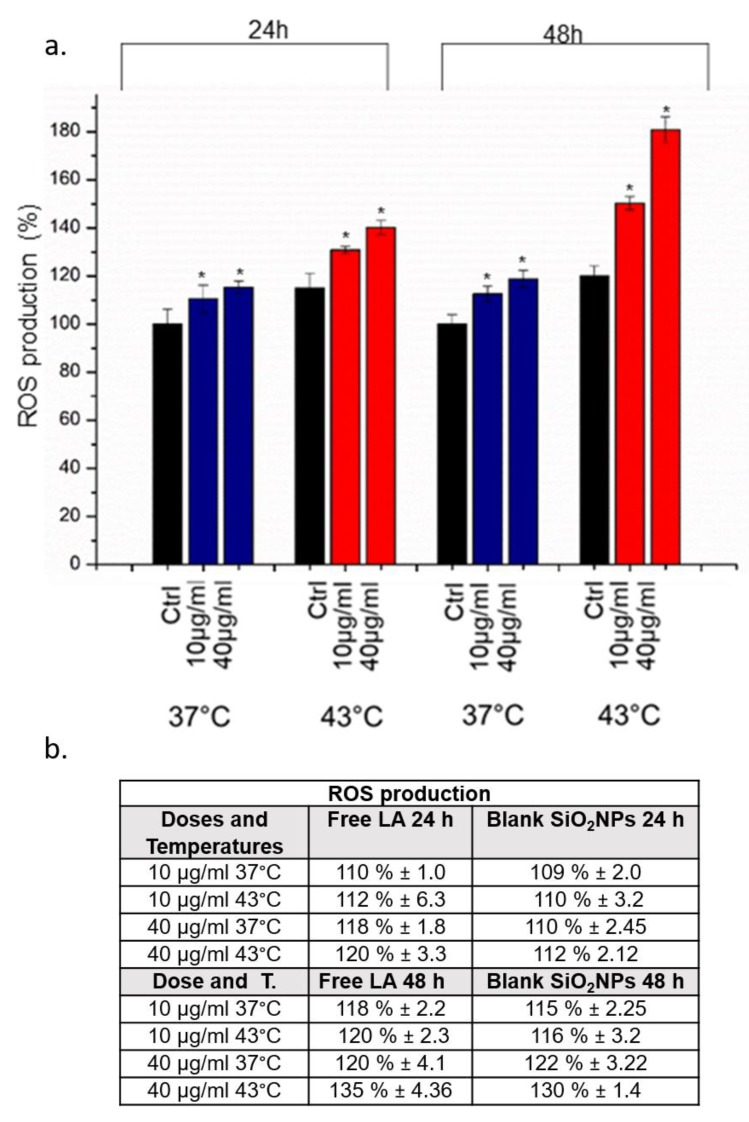
**a**. Effect of SiO_2_@LA NPs on the reactive oxygen species (ROS) level in MCF-7 cells. Cells were treated with of 10 µg/mL and 40 µg/m of NPs for 24 h and 48 h at 37 °C and 43 °C incubated with 100 µM DCFH-DA. Cells fluorescence was measured. Controls sample were represented by cells without SiO_2_@LA NPs exposure. As a positive control (P), cells were incubated with 500 µM H_2_O_2_ showing a ca. 300% DCFH-DA increase (not show). **b**. ROS values expressed in percentage obtained on MCF-7 cells after 24 h and 48 h exposure to 10 µg/mL and 40 µg/mL of blank SiO_2_NPs and free LA at 37 °C and 43 °C. Data reported as mean ± SD from three independent experiments are considered statistically significant compared with control (*n* = 8) for *p* value ˂ 0.05, (<0.05 *).

We also observed ability of SiO_2_@LA NPs to induce oxidative stress through ROS production by DCFH-DA assay at 10 µg/mL and 40 µg/mL (24 h and 48 h). Also in this case, the strong ROS production was observed when the temperature was increased to 43 °C in comparison to cells exposed to NPs and 37 °C: as showed in Figure 6, after 48 h the percentage of ROS increased up to 180% at the higher concentration tested.

**Figure 7:**
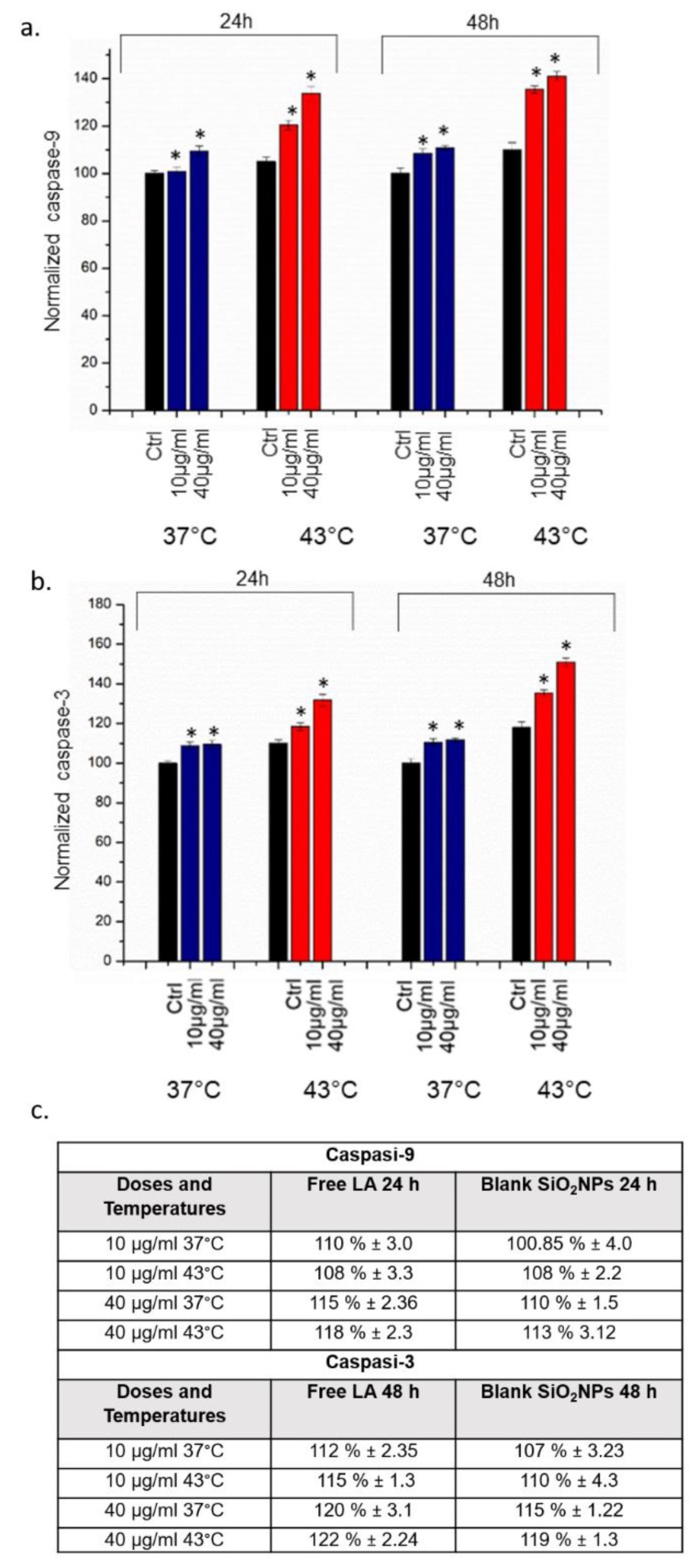
**a**,**b** Effect of SiO_2_@LA NPs on levels of apoptosis. Caspase-9 and Caspase-3 assay was performed incubating MCF-7 with of 10 µg/mL and 40 µg/m of NPs for 24 h and 48 h at 37 °C and 43 °C. Controls were represented by cells without SiO_2_@LA NPs exposure. **c.** Caspases 3–9 levels values obtained incubating MCF-7 with free LA and blank SiO_2_NPs at 10 µg/mL and 40 µg/m of NPs for 24 h and 48 h at 37 °C and 43 °C. Data are reported as mean ± SD from three independent experiments are considered statistically significant compared with control (*n* = 8) for *p* value ˂ 0.05 (<0.05 *).

The apoptosis and inflammation phenomena were also triggered by the activity of caspases, a family of cysteine proteases (Figure 7). We analysed the caspase-9 (initiator) and caspase-3 (executioner) activation after exposure to 10 µg/mL and 40 µg/mL of SiO_2_@LA NPs for 24 h and 48 h. Also in this case, the control samples were represented with cells treated only with the two temperatures selected for this study (37 °C and 43 °C). Cells with NPs inside, once exposed to temperature of 43 °C, underwent far greater caspases activation than cells exposed to 37 °C: at the higher concentration tested, both caspase-3 and caspase-9 increased up to 145% to 155% respectively. These effects were higher than the use of high temperature alone.

**Figure 8:**
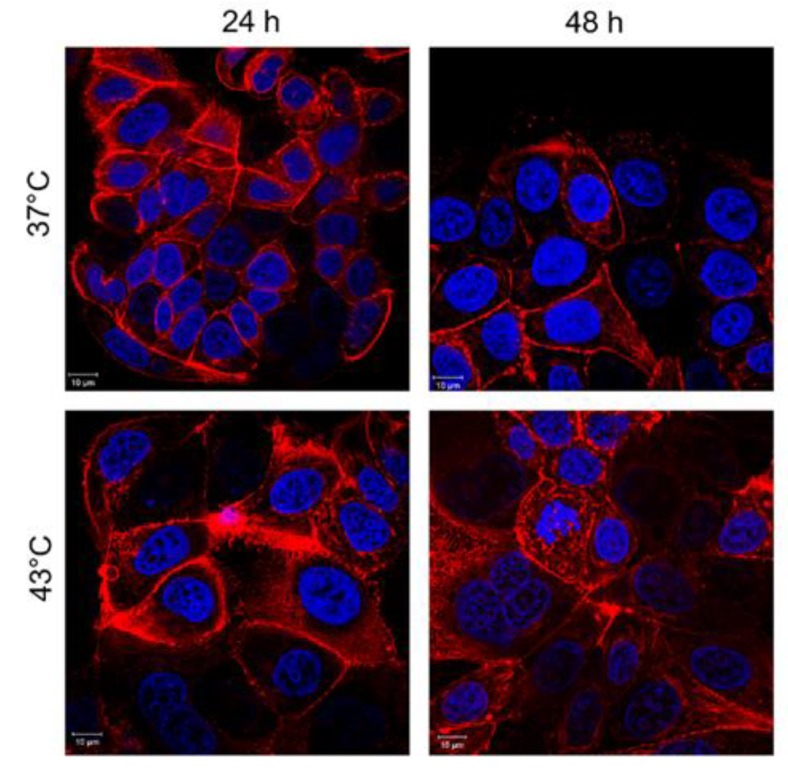
Effects of temperature on actin network of MCF-7 cells after 24 h and 48 h. Cells were fixed and then stained with Phalloidin–ATTO 488 and DAPI. The 2D images of cortical actin were acquired by a Zeiss LSM700 (Zeiss) confocal microscope equipped with an Axio Observer Z1 (Zeiss) inverted microscope using a ×100, 1.46 numerical aperture oil immersion lens. All data were processed by ZEN software (Zeiss).

Confocal microscopy analyses of MCF-7 incubated with 10 µg/mL and 40 µg/mL of SiO_2_@LA NPs for 24 h and 48 h were reported. The changes in cell morphology were another proof to confirm the combined action on MCF-7 cells. The cells exposed only to heat treatment presented negligible ruffles in some cell regions (Figure 8).

**Figure 9:**
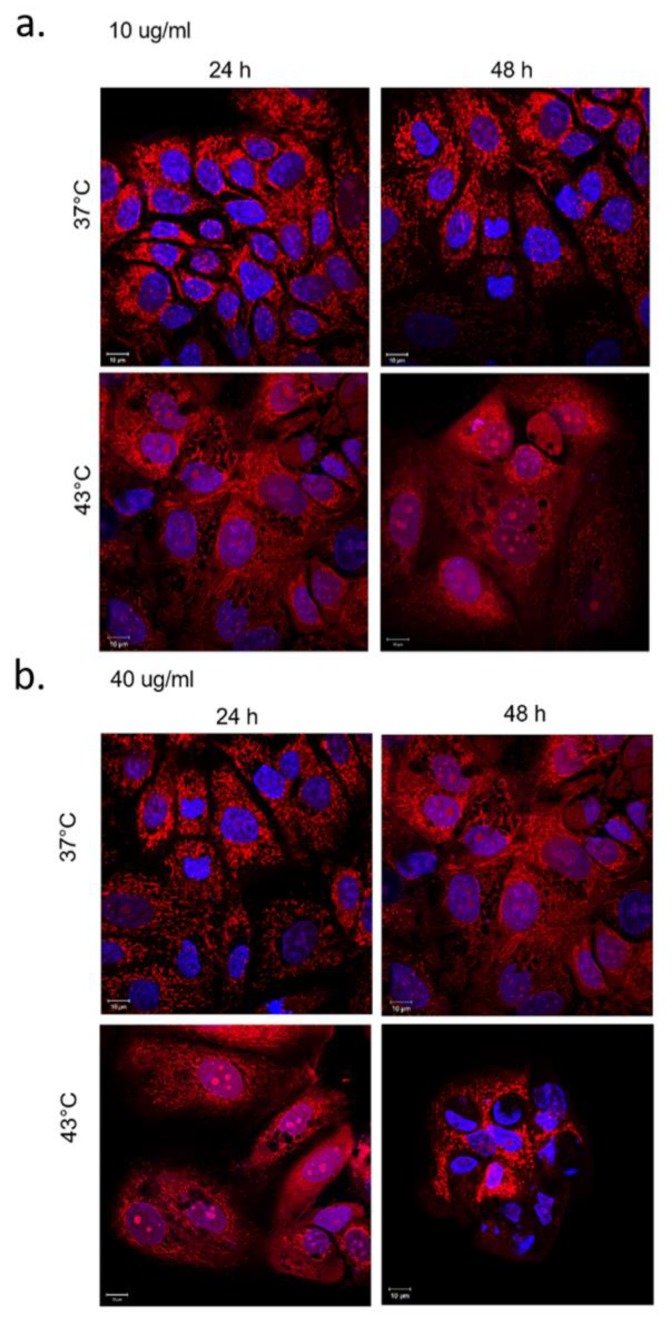
Effects of SiO_2_@LA NP_S_ and temperature on actin network and nuclei of MCF-7 cells. MCF-7 were treated with 10 µg/mL (**a**) and 40 µg/mL (**b**) of NPs for 24 h and 48 h, fixed and then stained with Phalloidin–ATTO 488 and DAPI. The 2D images of cortical actin were acquired by a Zeiss LSM700 (Zeiss) confocal microscope equipped with an Axio Observer Z1 (Zeiss) inverted microscope using a ×100, 1.46 numerical aperture oil immersion lens. All data were processed by ZEN software (Zeiss).

When the cells underwent the synergic effects of temperature and LA release as consequence of its fusion at melting temperature (43 °C), MCF-7 changed their morphology from cobblestone-like to a more spindle-like appearance. Indeed, the MCF-7 showed significant changes of the actin pattern, particularly evident at 48 h after SiO_2_@LA NPs and 43 °C of treatment (Figure 9a,b).

**Figure 10:**
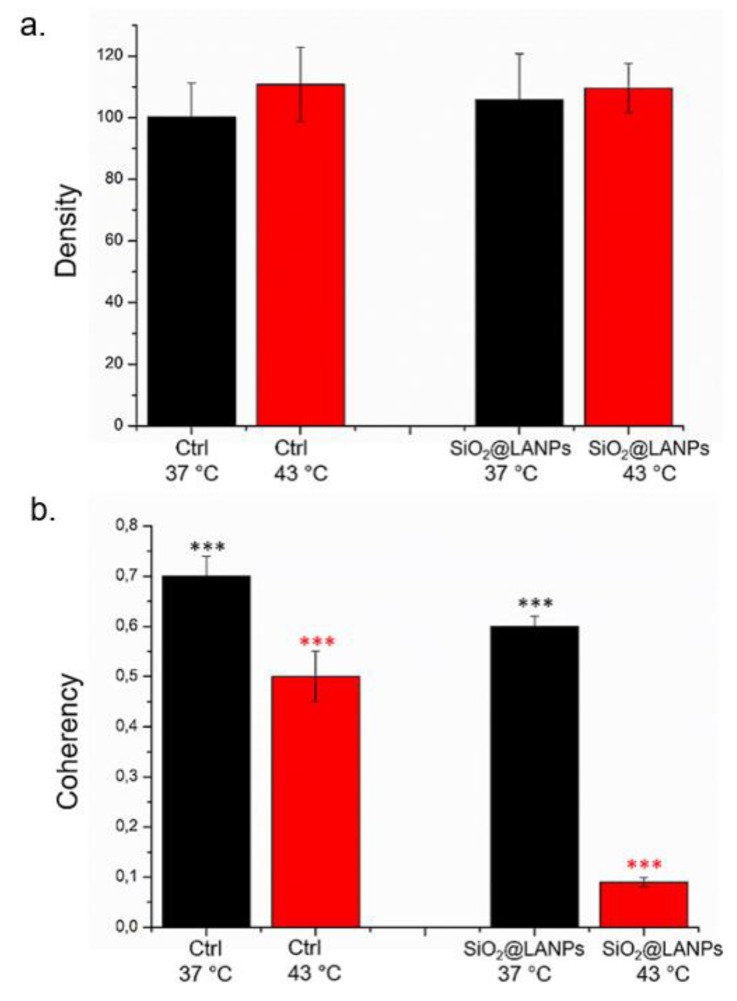
Integrated density (**a**) and coherency (**b**) for MCF-7 with 40 μg/mL of SiO_2_@LA NPs after 48 h. The integrated density and coherency values were expressed as a mean value and relative SD, calculated from confocal acquisitions by ImageJ (calculation on 15 cells). The mean values and their standard deviations were reported in the histograms. Results were statistically significant for *p* < 0.05 (0.005***).

The quantification of actin network organization after SiO_2_@LA NPs exposure with and without temperature treatment (48 h of 40 μg/mL of SiO_2_@LA NPs) was evaluated by fluorescence density and coherency using ImageJ software that quantify the amount of actin and the degree of fibers orientation respectively. The fluorescence density of actin was not perturbed by thermal and NPs treatment. Untreated MCF-7 cells at 37 °C presented a density value of (100.2 ± 11): this remained almost unaffected upon NP treatments at 40 μg/mL (105.8 ± 15). When the thermal treatment was applied at 43 °C, also the density of actin-stained network remained the same before and after NPs treatment: (110.77 ± 12) for negative control and (109.6 ± 8) after SiO_2_@LA NPs exposure (Figure 10a). A different reorganization of actin was recorded by coherency analysis. At 37 °C, the coherency values of treated cells for SiO_2_@LA NPs treatment (0.6 ± 0.03) decreased with respect to the control (0.7 ± 0.04); the same trend, but more strong, was measured after thermal treatment: (0.4 ± 0.02) for control and (0.09 ± 0.03) for cells treated with NPs and heat (Figure 10b). These results clearly showed the significant changes of actin network as fibers orientation that did not alter the quantity of total actin expressed.

Another detectable phenomenon is the nuclei alterations in terms of circularity and roundness that are apoptosis indices [42], using ImageJ software as showed in Table 2.

For circularity, the values near 1.0 represent a perfect circle without roughness, whereas 0 represent an increase of asperity. So that, NPs induced in cells a reduction in nuclei circularity in a time and dose dependent manner. In addition, the effect of the high temperature increased this phenomenon. For example, after the combined treatment (NPs and 43 °C) at the higher concentration tested, the nuclear circularity value decreased to (0.69 ± 0.02), compared to the control (0.89 ± 0.02). Roundness represents the shape of nuclei. An increase of these values after NP_S_ and temperature treatment indicated rounder nuclei, which was associated with apoptosis induction. The enhancement of adverse effects in cells combining high temperature and NPs exposure can be explained because the heat treatment promoted cell perfusion, simplified the uptake of LA through the cell membrane together with morphological changes that augmented the cell area inducing more absorption of LA [43]. In addition, the oxidative stress and caspase activation induced by LA promoted the apoptosis event on MCF-7 that, combined with sub-lethal heat treatment, promoted cancer cell death presumably due to destruction of DNA repair mechanisms [44].

## 3. Materials and Methods

### 3.1. Synthesis of Core/Shell SiO_2_@LA NPs

The synthesis was carried out adopting the so-called Stöber method, following the procedure described in Stöber et al. [45] with some modifications in order to encapsulate PCMs materials as reported in [16]. An amount of LA (MW: 200.32 g/mol) was dissolved in 5 mL of ethanol to obtain 1 mM of LA solution in final volume of reaction. To ethanol-LA solution was added TEOS (100 µL), milliQ water (20 mL) and NH_4_OH solution (28.0–30.0%, 10 mL) for 2 h at 25 °C in order to maintain the LA under the melting temperature value. Acetone was used to block the reaction and the solution was centrifuged at 4000 rpm for 20 min. The SiO_2_@LA NPs were rinsed with a mix with 1:1 milliQ water and ethanol 5 times and successively dried under reduced pressure and then at 100 °C for 2 h to obtain a white nano-powder. The yield for this synthesis was about 80%.

### 3.2. Nanoparticles Characterizations (TEM, SEM, EDS, DLS, DSC)

Transmission electron microscope (TEM) characterizations were carried out with a JEOL Jem 1011 microscope, operating at an accelerating voltage of 100 kV (JEOL USA, Inc, Peabody, MA, USA). TEM samples were prepared by dropping a dilute solution of NPs in water on carbon-coated copper grids (Formvar/Carbon 300 Mesh Cu). Microscopy observations were made by means of a Scanning Electron Microscope (SEM, JEOL JSM-6480LV operating at an accelerating voltage of 20 kV, JEOL USA, Inc, Peabody, MA, USA), whereas, Energy-Dispersive X-ray Spectroscopy (EDS) were recorded with a Phenom ProX microscope (Phenom-World B.V., Eindhoven, Germany), at an accelerating voltage of 10 kV. The samples were prepared by dropping a solution of NPs in water on mono-crystalline silicon wafer. Dynamic Light Scattering (DLS) and ζ-potential measurements were performed on a Zetasizer Nano-ZS equipped with a 4.0 mW HeNe laser operating at 633 nm and an avalanche photodiode detector (Model ZEN3600, Malvern Instruments Ltd., Malvern, UK). Measurements were made at 25 °C in aqueous solutions (pH 7). Thermal properties of SiO_2_@LA NPs, such as melting temperature (Tm), cooling temperatures (Tc) and enthalpy (Δhm), were measured by means of a DSC instrument (Mettler Toledo 822, Greifensee, Switzerland). The analysis was performed on dried samples under a constant stream of nitrogen (60 mL/min) at atmospheric pressure, applying a first isothermal step at −10 °C for 5 min, followed by a heating scan between −10 °C and 100 °C at 1 °C/min. Then, the samples were submitted to a further isothermal step at 100 °C for 5 min, followed by a cooling scan from 100 °C to −10 °C at 1 °C/min. The phase change temperatures (melting and freezing points) were evaluated as the intersection between the tangent to the maximum rising slope of the peak and the sample baseline.

#### 3.2.1. LA Loading in SiO_2_NPs

To determine the loading of LA in the SiO_2_NPs, we used the rotary evaporation to dry 5 mL of SiO_2_@LA NPs. After this step, sample were dissolved in methanol and chemically deriving with naphthacyl ester. The obtained product was analysed by reversed-phase high performance liquid chromatography (HPLC, ThermoFisher, Waltham, MA, USA). The solvent gradient of methanol/acetonitrile/water was changed from 80:10:10 (*v/v/v*) to 90:10:0 (*v/v/v*). The temperature of column (4 µm, 4.6 mm× 250 mm, Waters, Milford, MA, USA) was maintained at 30 °C. The derived LA was detected by Cary 300 UV–vis spectrophotometer (Varian, Palo Alto, CA, USA) at a resolution of 1 nm using a 5 mm path length quartz cuvettes, at a wavelength of 250 nm (20 °C). The LA concentration was obtained using the following equation (derived from calibration curve of free LA)

LA loading content (%) = Amount of LA in SiO_2_NPs/Amount of SiO_2_@LA NPs × 100% (2)

#### 3.2.2. Thermo Sensibility of SiO_2_@LA NPs

In vitro LA release at different temperatures was detected using 10 µg/mL and 40 µg/mL of SiO2@LA NPs dispersed in water. We applied different temperatures (37 °C, 39 °C, 41 °C, 43 °C, 45 °C, 47 °C) to the SiO_2_NPs@LA solutions in order to occur the thermo sensibility of novel SiO_2_@LA NPs. After temperatures treatment, NPs were ultracentrifuged at 14,000 rpm and the surnatant was analysed by UV/VIS at 250 nm of wavelength.

### 3.3. Cell Culture

MCF-7 cells were maintained in high glucose DMEM with 50 μM of glutamine, supplemented with 10% FBS, 100 U/mL of penicillin and 100 mg/mL of streptomycin. Cells were incubated in a humidified controlled atmosphere with a 95% to 5% ratio of air/CO_2_, at 37 °C.

### 3.4. Uptake of SiO_2_@LA NPs

10^5^ cells were seeded in 1 mL of medium in each well of a 6-well plate. After 24 h at 37 °C, the medium was replaced with fresh medium containing the SiO_2_@LA NPs at concentrations of 10 µg/mL and 40 µg/mL. After 24 and 48 h of incubation at 37 °C, the medium was removed; the cells were washed three times with PBS (pH 7.4), trypsinized, and counted using a cell-counting chamber. 3.6 × 10^5^ cells were suspended in 200 µL of milliQ and treated with HCl/HNO_3_ 3:1 (*v/v*), diluted to 5 mL and analysed to evaluate Si content. Elemental analysis was carried out by ICP-AES with a Varian Vista AX spectrometer (Varian, Palo Alto, CA, USA).

### 3.5. Hyperthermia Treatment

Hyperthermal stress was induced increasing the incubator temperature to 43 °C as previous reported in [46]. After 45 min of exposure, cells were reported at temperature of 37 °C for 12 h. In this way, the assays reported and the confocal images were conducted in heat treated cells and without heat exposure.

### 3.6. MTT and ROS Production

MCF-7 cells were seeded in 96 well micro-plates at concentration of 5 × 10^3^ cells/well after a 24 h of stabilization of the cells. NPs suspension stock solution was added to the cell media in order to obtain final concentrations of 10 µg/mL and 40 µg/mL for 24 and 48 h. At the end of exposure, standard WST-8 assay (Sigma) was used to test viability both of microplates at 37 °C and micro-plates after a heating at 43 °C for 45 min following the hyperthermia treatment reported above. Viability test was also performed in cells heated at 43 °C without NPs exposure during 24 and 48 h. Assays were performed following the procedure previously described in De Matteis et al. [47]. The same procedure was used to test the possible reduction of viability induced by blank SiO_2_NPs and free LA (used at the same concentration (10 µg/mL and 40 µg/mL) for 24 and 48 h on MCF-7. Data were expressed as mean ± SD. Differences in cell proliferation (WST-8) between cells treated SiO_2_@LA NPs and the control were considered statistically significant performing a *t*-student test with a *p-*value ˂ 0.05.

DCF-DA (2′,7′-dichlorofluorescein diacetate, Sigma) assay was performed on MCF-7 cells to measure the levels of ROS. MCF-7 cells were seeded in 96-well micro-plates and treated with NPs at a final concentration of 10 µg/mL and 40 µg/mL. After 24 and 48 h of cells–NPs interaction the assay was performed onto micro-plates following the procedure reported by De Matteis et al. [48]. The same procedure was used to test the ROS production induced by blank SiO_2_NPs and free LA using the same concentration (10 µg/mL and 40 µg/mL) for 24 and 48 h on MCF-7. Data were expressed as mean ± SD. Differences in ROS generation between cells treated with SiO_2_@LA NPs and controls were considered statistically significant performing a student t test with a *p*-value < 0.05.

### 3.7. Caspase-3 and Caspase-9 Induction

Caspase-3 activity was performed by using CASP-3-C assay kit (Sigma-Aldrich). MCF-7 cells (1 × 10^7^ cells/mL) were treated for 24 and 48 h with SiO_2_@LA NPs (10 µg/mL and 40 µg/ mL). After NPs exposure, cells were lysed on ice with 50 µL of lysis buffer for 10 min following the addition of 50 µL of reaction buffer containing dithiothreitol (DTT) and 5 µL of caspase-3 substrate solution (Acetyl-Asp-Glu-Val-Asp p-nitroanilide [Ac-DEVDpNA]). The caspase-3 activity of each sample was expressed in μMol of pNA released per min per mL of cell lysate; the percentage of caspase-3 activity was calculated control × 100. Caspase-9 activity was analyzed by using Caspase-9 Assay Kit (Abcam, Cambridge, UK). MCF-7 (5 × 10^6^) cells were incubated with SiO_2_@LA NPs (10 µg/mL and 40 µg/mL) at two time points (24 and 48 h). Successively, cells were lysed with 50 µL of lysis buffer for 10 min, following the addition of 50 µL of reaction buffer containing dithiothreitol (DTT) and 5 µL of caspase-9 substrate solution Leu-Glu-His-Asp (LEHD) peptide (LEHD-p-NA). The caspase-9 activity of each sample was expressed in μMol of pNA released per min per mL of cell lysate; the percentage of caspase-9 activity was calculated control × 100. Fluo Star Optima (BMG LABTECH, Ortenberg, Germany) was used to measure the pNA released at 450 nm for caspase-3 and 405 nm for caspase-9 and data analyzed with MARS Data Analysis Software (BMG LABTECH, Ortenberg, Germany). The same procedure was used to test the ability of blank SiO_2_NPs and free LA to induce caspases-3 and 9 activation using the same concentrations (10 µg/mL and 40 µg/mL) for 24 and 48 h on MCF-7

### 3.8. Confocal Measurements

MCF-7 cells were seeded in glass bottom Petri dishes (Corning) at 10^5^ cells/well of concentration. After 24 h of stabilization, cells were exposed to SiO_2_@LA NPs (10 µg/mL and 40 µg/mL) for 24 and 48 h; at the end of incubation time, the medium supplemented with NPs was removed and cells washed three times with Phosphate Buffered Saline (PBS). In order to test the effect of heating on cell actin another set of cells replicates was conducted incubating cells with SiO_2_@LA NPs for 48 h and successively heated at 43 °C for 40 min. At the end of this procedure DMEM with NPs was removed and cells washed three times with PBS. F-Actin and organelles were marked in living cells. In particular, for actin staining, we used Cell Light™ Actin-GFP, BacMam 2.0 (Thermo-Fisher Scientific) adding 2 μL of dye reagent per 10^3^ cells in media for 16 h at 37 °C. All confocal images were acquired using LSM700 (Zeiss, Germany) confocal microscope mounted on an Axio Observer Z1 (Zeiss, Germany) inverted microscope, by using the Alpha Plan-Apochromat (Zeiss) 100 × oil-immersion with 1.46 NA. The acquisitions were conducted by ZEN2010 (ZEISS, Germany) and nuclear morphometric alterations were evaluated on 20 cells for each sample by means ImageJ 1.47 analysis software (Bethesda, Maryland, USA) in terms of circularity and roundness. Circularity is a shape descriptor that quantified, as closely the shape of the investigated object is comparable to a circle: its value ranges from 0 to 1 (perfect circle). Roundness parameter is similar to circularity but it is independent to local irregularity on surface particle. Integrated density and coherency of F-actin were performed on 15 cells using OrientationJ plugin by choosing a specific area in confocal images. Value of orientation and coherency represented the degree of actin fibers orientation: values near 0 represented disordered fibers whereas value of about 1 represented fibers perfectly aligned ones show coherency. The sum of the pixels values in a specific region of confocal acquisitions represented the integrated density.

## 4. Conclusions

Today, the development of new strategies for the treatment of some types of cancer, focuses on the use of green extracts as a potential alternative to synthetic molecules in order to establish if they can reduce—as much as possible—the toxic effects that characterize the use of traditional chemotherapeutic drugs. Thermotherapy is a technique that exploits the physical principle of heat. By combining the heat treatment, which operates at a temperature of about 43 °C, with the use of encapsulated LA (having pro-apoptotic/ inflammatory effects and a melting temperature of 43 °C) in a silica biocompatible shell, we have obtained satisfactory results in breast cancer cells, which represent a “proof of concept” for the design of new thermo-responsive nanomaterials as anticancer agents.

## Figures and Tables

**Table 1:** Concentration value of lauric acid (LA) in SiO_2_NPs expressed in µg/mL (obtained with HPLC analysis) and concentration of SiO_2_@LA NPs expressed in mg/mL. The resulting LA loading content expressed in (%) was obtain applying the equation: amount of LA in SiO_2_NPs/Amount of SiO_2_@LA NPs × 100%**.**

	Concentration of LA in SiO_2_NPs (µg/mL)	Concentration of SiO_2_@LA NPs (mg/mL)	LA Loading Content (%)
SiO_2_@LA NPs	281.25 ± 5.78	2.67 ± 0.7	10.5 ± 1.35

**Table 2:** Nuclear circularity and nuclear roundness measured by ImageJ software. The control samples were MCF-7 exposed to 37 and 43 °C for 24 h and 48 h without SiO_2_@LA NPs exposure. The treated samples were represented by cells exposed to 10 μg/mL and 40 μg/mL for 24 h and 48 h and 37 °C and 43 °C.

		Nuclear Circularity	Nuclear Roundness
CTRL	24 h, 37 °C	0.91 ± 0.20	0.80 ± 0.05
	48 h, 37 °C	0.90 ± 0.01	0.79 ± 0.02
	24 h, 43 °C	0.89 ± 0.03	0.79 ± 0.04
	48 h, 43 °C	0.89 ± 0.02	0.78 ± 0.02
10 µg/mL	24 h, 37 °C	0.89 ± 0.04	0.81 ± 0.03
	48 h, 37 °C	0.85 ± 0.02	0.79 ± 0.09
	24 h, 43 °C	0.87 ± 0.07	0.83 ± 0.01
	48 h, 43 °C	0.80 ± 0.02	0.85 ± 0.04
40 µg/mL	24 h, 37 °C	0.88 ± 0.02	0.80 ± 0.04
	48 h, 37 °C	0.75 ± 0.04	0.83 ± 0.05
	24 h, 43 °C	0.80 ± 0.01	0.87 ± 0.03
	48 h, 43 °C	0.69 ± 0.02	0.88 ± 0.05
